# Supplementary material for: Water extract of ginseng alleviates parkinsonism in MPTP–induced Parkinson’s disease mice
Source: PLoS One. 2024 Sep 20;19(9):e0296424. doi: 10.1371/journal.pone.0296424 (PMC11414931; doi:10.1371/journal.pone.0296424)

the manuscript's main figures

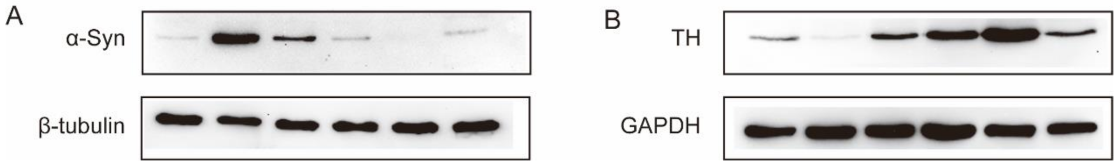

Original image of Western Blot

Original blot of α-Syn

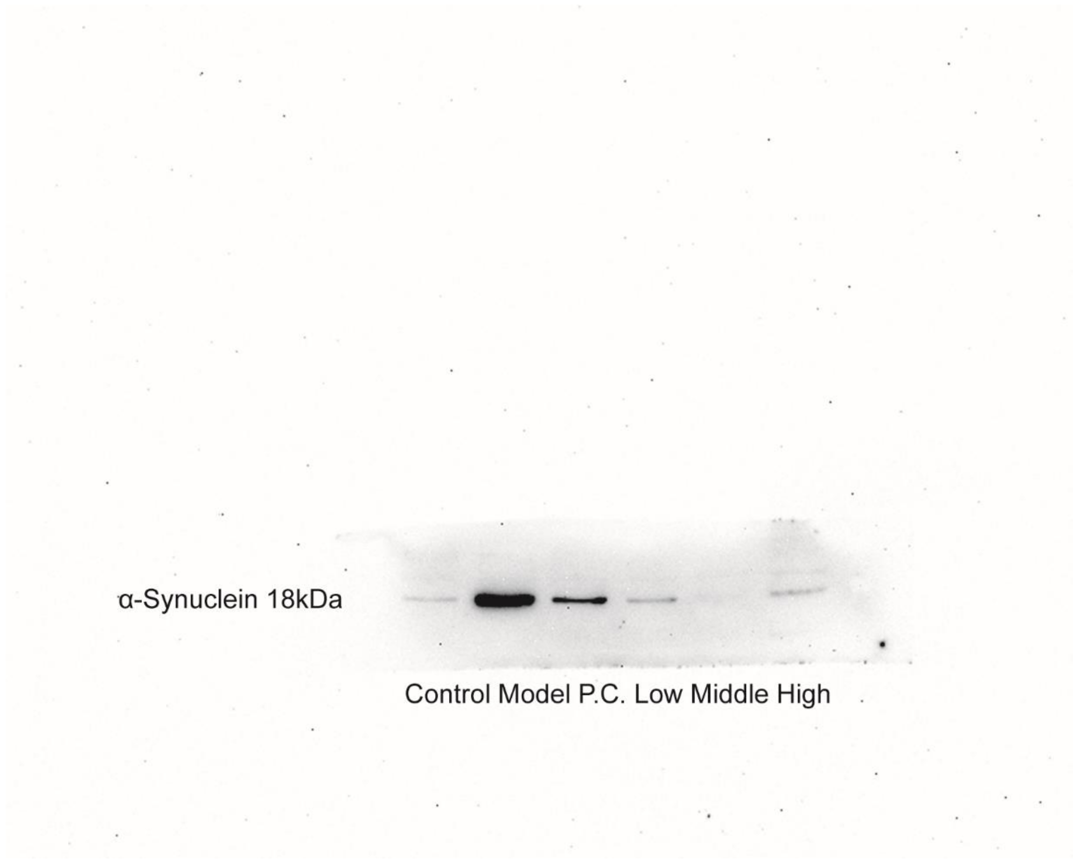

Supplemental figures of α-Syn

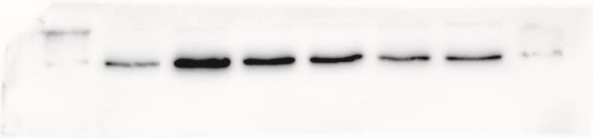

α-Synuclein 18kDa

Control Model P.C. Low Middle High

α-Synuclein 18kDa

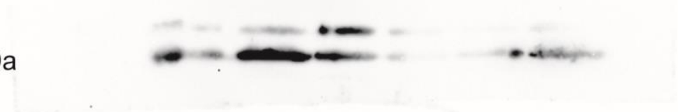

Control Model P.C. Low Middle High

Original blot of β-Tubulin

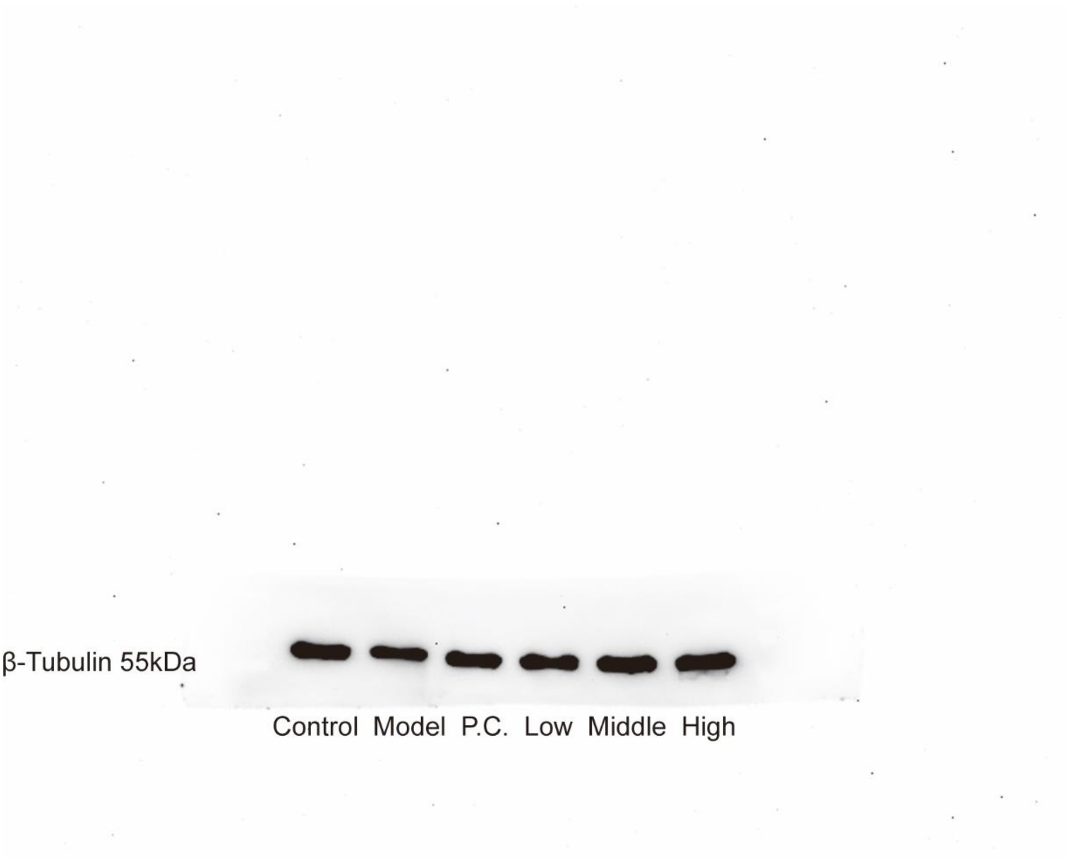

Supplemental figures of β-Tubulin

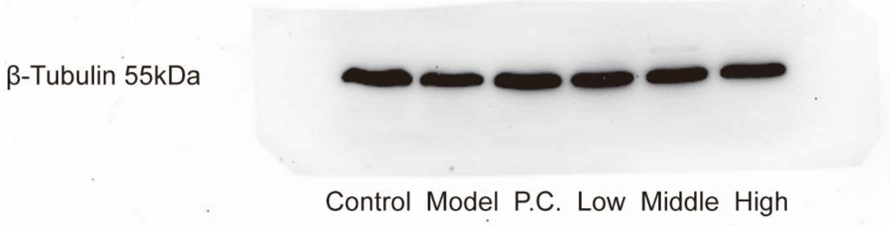

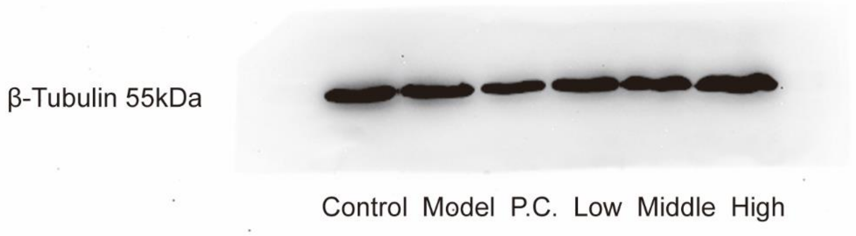

Original blot of TH

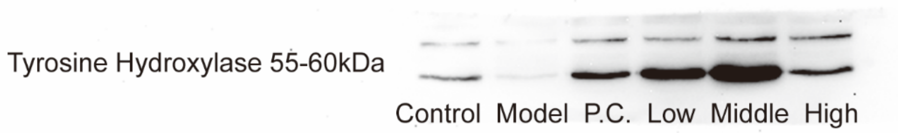

Supplemental figures of TH

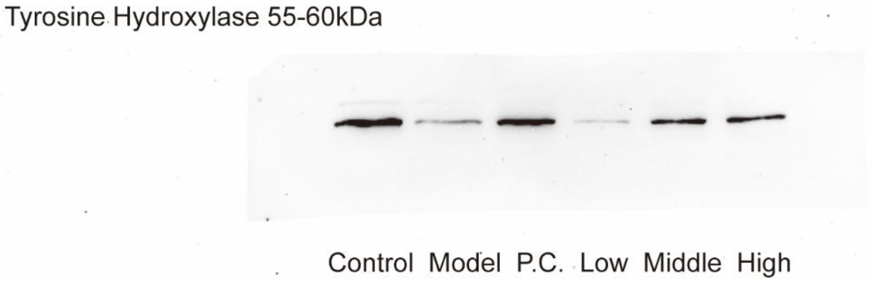

Tyrosine Hydroxylase 55-60kDa

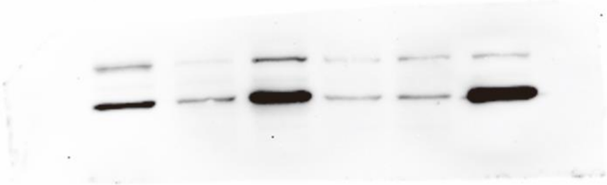

Control Model P.C. Low Middle High

Original blot of GAPDH

GAPDH 36kDa

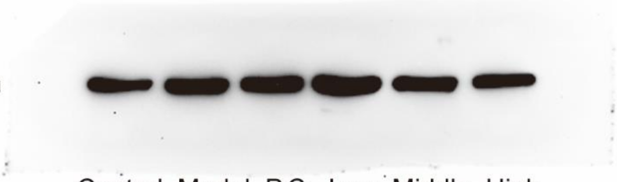

Control Model P.C. Low Middle High

Supplemental figures of TH

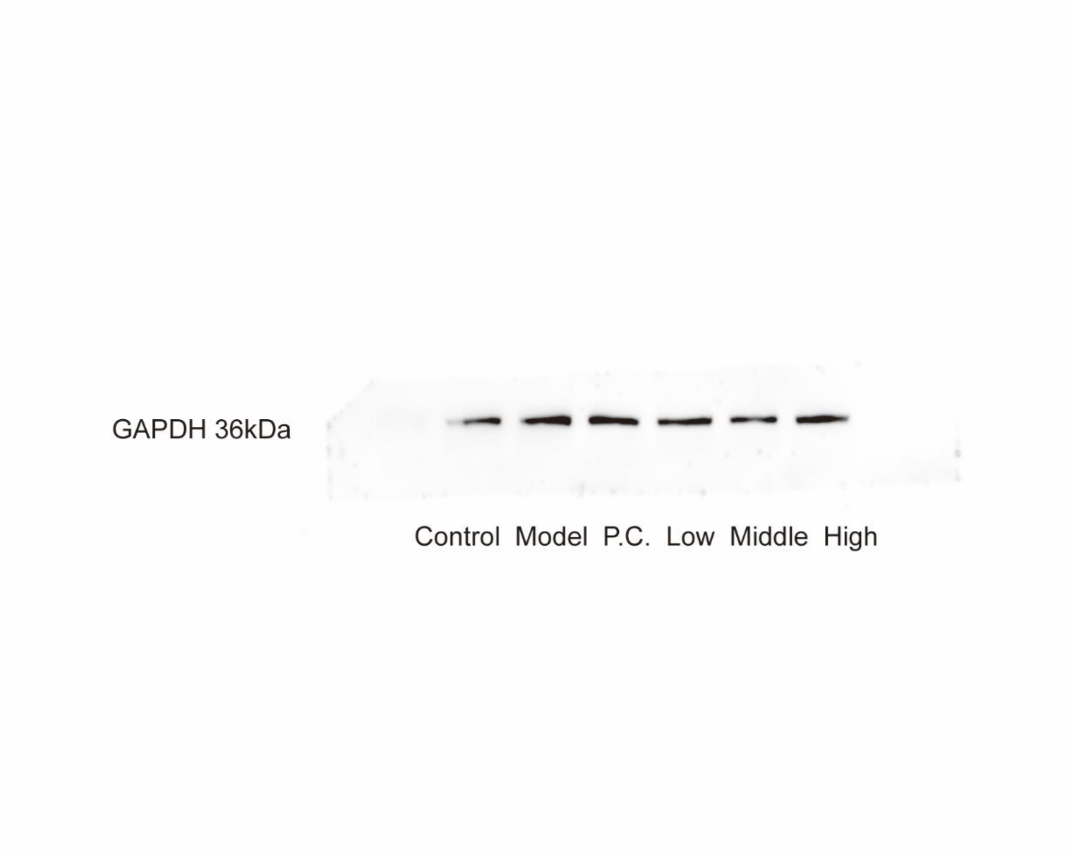

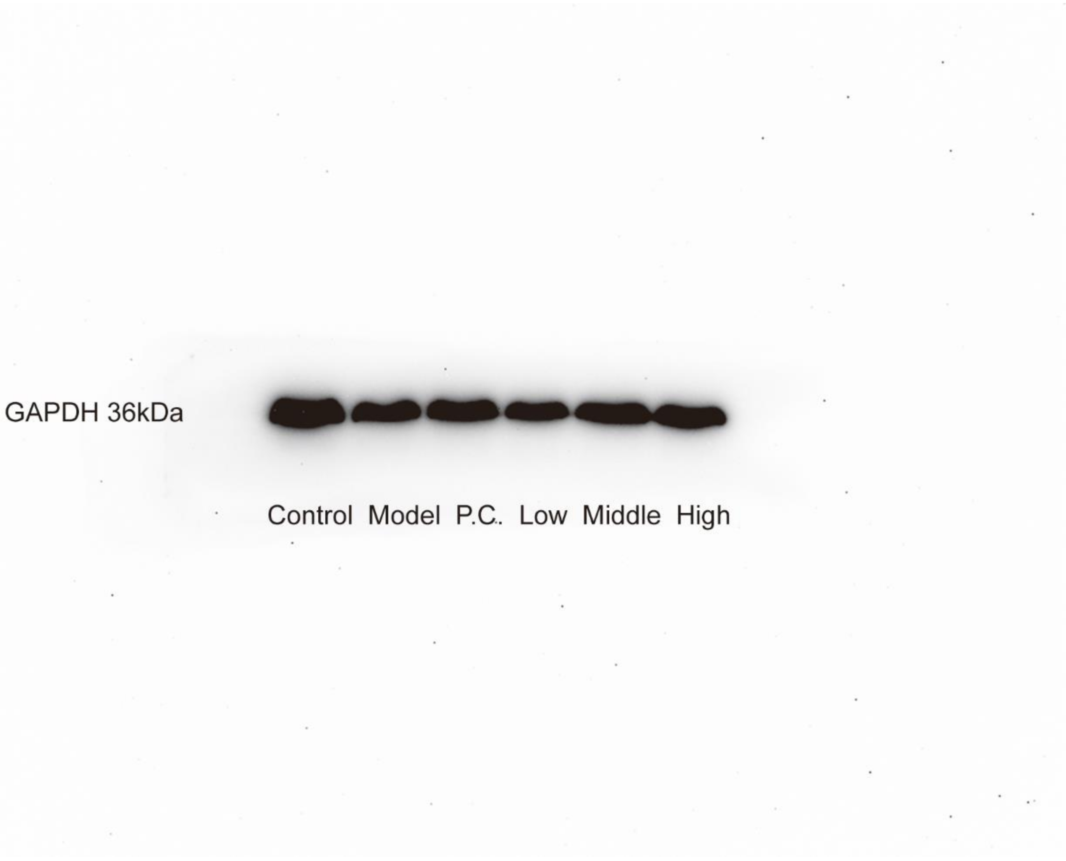

Supplement: S1 Raw images — (PDF) [file pone.0296424.s001.pdf]
